# Glocalizing AfriMEDS: a decolonial, Ubuntu-grounded roadmap for operationalizing global competency standards in African medical education

**DOI:** 10.3389/fmed.2026.1859848

**Published:** 2026-08-18

**Authors:** Nathaniel Mofolo, Priscilla Mpho Jama, Gina Wisker

**Affiliations:** 1 Department of Family Medicine, School of Medicine, Faculty of Health Sciences, University of Pretoria, Pretoria, South Africa; 2 School of Clinical Medicine, Faculty of Health Sciences, University of the Free State, Bloemfontein, South Africa; 3 School of Management, University of Bath, Bath, United Kingdom; 4 Division of Student Learning and Development, Faculty of Health Sciences, University of the Free State, Bloemfontein, South Africa

**Keywords:** African Indigenous Health Systems, AfriMEDS, competency-based medical education, decolonization, faculty development, Global South, glocalization, Ubuntu

## Abstract

**Background:**

Training community-responsive physicians for South Africa’s under-resourced rural and peri-urban settings remains a defining challenge for African medical education. The AfriMEDS framework, adopted in 2014, succeeded in establishing a shared national competency vocabulary and in standardizing the technical, medical-expert foundations of undergraduate training; it has not yet, however, authentically integrated Indigenous Knowledge Systems (IKS), Ubuntu ethics, indigenous-language proficiency, or formal partnership with Traditional Health Practitioners (THPs). Competency-Based Medical Education (CBME) implementation, while nationally mandated, remains compliance-oriented rather than genuinely glocalized.

**Objectives:**

To re-examine documented findings from an existing AfriMEDS evaluation, identify where authentic glocalization is missing, and translate those deficits into an integration framework and transferable transformation roadmap.

**Methods:**

A three-stage interpretive secondary re-analysis engaged the published outputs of a prior mixed-methods doctoral study at the University of the Free State (UFS) MBChB programme, drawing on survey data from 71 medical interns, interviews with 15 educators, and analysis of six curriculum documents. A decolonial, Ubuntu-informed lens re-synthesized the documented findings onto five AIHS pillars. The resulting framework and roadmap are conceptual and inferential; future community co-validation is required.

**Results:**

The glocalization gap is both empirical and epistemic. Intern self-reported adequacy fell below the 80% threshold the highest reaching 73.2% for five AfriMEDS roles: Leader-Manager (63%), Community-Based Education (71%), Collaborator (72%), Health Advocate (78%), and Communicator (79%); the Medical Expert role scored highest at 91%. Only 40% of interns felt adequately prepared in indigenous-language communication. All community-based education competency items fell below the 80% threshold. IKS, THP protocols, and Ubuntu ethics were absent or tokenistic in all six institutional documents. Educators identified staff shortages and inadequate CBME teaching preparation as principal structural barriers. The AIHS Integration Framework comprises five pillars: epistemic pluralism, community partnership pedagogy, contextualized assessment, educator capacity building, and decolonial quality assurance, operationalized through a six-step roadmap.

**Conclusion:**

Glocalizing AfriMEDS is a deliberate move from policy compliance toward epistemic freedom. The proposed framework demonstrates that CBME can be simultaneously globally rigorous and locally pluriversal, positioning indigenous knowledge as a necessary complement to, rather than a replacement for, the biomedical core. It offers African medical schools, the Health Professions Council of South Africa (HPCSA), and Global South partners a transferable roadmap for producing graduates who are globally informed and communally accountable. Phase 2 validation through community co-design, Delphi consensus, and multi-site piloting is proposed as future work.

## 1 Introduction

The tension between globally endorsed competency standards and the socio-cultural, linguistic, and health-system realities of physicians’ practice environments increasingly defines Health Professions Education (HPE) (1). Robertson (2) theorized glocalization as the simultaneous globalization and localization of social practices; Roudometof (3) elaborated it as foundational to curriculum reform and workforce development in the Global South (4, 5). In medical education, the aspiration is clear: graduates must meet internationally recognized benchmarks while remaining deeply rooted in the communities they serve. Direct transplantation of Western-derived frameworks without interrogating their epistemic underpinnings can reproduce colonial hierarchies of knowledge, particularly where Indigenous Knowledge Systems (IKS); culturally embedded ways of knowing and healing, are marginalized (6, 7).

In South Africa, this tension is amplified by the legacies of apartheid, significant health disparities, and the National Health Insurance Policy. In 2014, the HPCSA adopted AfriMEDS (27); a regional CanMEDS adaptation explicitly aspiring to embed community-based education (CBE) and community-oriented primary care (COPC) through seven physician roles (27, 28). AfriMEDS is adapted from the CanMEDS Physician Competency Framework of the Royal College of Physicians and Surgeons of Canada (28), and the Royal College is acknowledged throughout this article as the source of that framework.

*It is important to state plainly what this adoption achieved, because the critique that follows depends on it. AfriMEDS was a substantive and necessary advance. It gave South African medical education a common competency language, embedded social accountability in national accreditation discourse, and, on the evidence re-examined here, succeeded in producing graduates who feel well prepared in the medical-expert, scholar, and professional roles that underpin safe acute and infectious-disease care*. That biomedical competence is not the problem to be solved; it is the achievement to be protected. Indigenous knowledge integration is advanced in this paper as a necessary complement to that competence, not as a substitute for it. The argument is therefore additive rather than oppositional: a graduate must be able to manage a child in septic shock *and* consult respectfully, in a shared language and an Ubuntu-relational register, with the family and the traditional practitioner who will care for that child after discharge.

Empirically, however, adoption has been primarily documentary: a mixed-methods doctoral evaluation at UFS and the subsequent Beyond Compliance study found marked gaps in the non-medical-expert roles most closely linked to community responsiveness (8, 9); See Table 1. IKS, THP collaboration protocols, and Ubuntu-aligned community ethics are absent from curricular architecture, making AfriMEDS a globally borrowed framework that still requires genuine localization. The curriculum ideologies underlying AfriMEDS have been critiqued as predominantly scholar-academic and efficiency-oriented rather than social-reconstructionist (10), directly corroborating the IKS-absence argument advanced in this paper.

**Table 1:** Non-duplication matrix.

Dimension	DBA thesis (8)	Beyond compliance (9)	Present manuscript
Primary purpose	Original empirical evaluation of AfriMEDS at UFS	Published report of doctoral empirical findings	Theory-guided secondary re-analysis; framework and roadmap
Analytical lens	CBME evaluation; mixed-methods	Mixed methods CBME outcomes reporting	Decolonial theory; Ubuntu; AIHS integration
New construct	Empirical findings	Published evidence base	AIHS Framework + six-step roadmap (not in either prior work)
Audience	University of Bath examiners	HPE researchers; CBME scholars	Curriculum committees; HPCSA; Global South HPE programmes
Overlap risk	Primary data source used	Primary findings reported	No raw data reused

This table clarifies the distinct contribution of this manuscript relative to the two prior outputs and addresses the salami-slicing concern.

Over 80% of South Africa’s population consults THPs, and indigenous languages remain central to health-seeking practices (11, 12). Ubuntu philosophy; *umuntu ngumuntu ngabantu,* offers a locally grounded ethical foundation for HPE (13–15). Glocalization and decolonization are related but distinct: glocalization foregrounds contextual relevance; decolonization demands epistemic justice. Authentic AfriMEDS glocalization is achievable only through a simultaneously decolonial lens grounded in Ubuntu and AIHS. There is therefore a critical need for an integrated, practically oriented roadmap that operationalizes AfriMEDS as an authentically glocal framework; global in its competency architecture, yet re-rooted in AIHS and Ubuntu ethics.

### 1.1 Study aim and objectives

The study aimed to develop a transferable conceptual framework and implementation roadmap that glocalize AfriMEDS by embedding AIHS and Ubuntu-informed principles into its design, assessment, and quality-assurance processes. Specifically: Re-examine documented findings from the original evaluation to identify where authentic glocalization is missing; Translate those deficits into a decolonial AIHS Integration Framework; and Operationalize the framework as a six-step AfriMEDS–AIHS implementation roadmap for HPCSA-accredited schools and other Global South programmes.

The practical destination of the paper is this roadmap, set out in Section 3.6 and summarized in Table 2; the analysis that precedes it serves to establish, in evidence, why each of its six steps is necessary.

**Table 2:** Six-step AfriMEDS–AIHS roadmap: pillar linkages, implementation components, and proposed outcome-level indicators.

Step	AIHS pillar	Key activity	AfriMEDS role(s)	Proposed indicator*	Benchmark*
1. Decolonial audit	Pillar 5	AIHS gap-analysis dashboard	All roles	% AIHS pillars in phase guides	≥80% within 24 mo*
2. Epistemic co-design	Pillars 1 & 2	THP glocalization working group	Communicator; collaborator; health advocate	IKS modules co-designed per phase	≥2 per phase/yr*
3. CBE & language restructuring	Pillar 2	THP co-facilitated placements; credited language modules	Communicator; health advocate	Credited language-module completion; THP-encounter journals	≥90%*
4. Faculty development	Pillar 4	Phased AIHS faculty development	All (educator-facing)	% faculty AIHS CPD annually	≥70% within 3 yr*
5. Summative assessment	Pillar 3	Community-diagnosis OSCE; portfolio rubric	Communicator; professional; collaborator	Indigenous-language OSCE pass rate	≥75% pass*
6. Decolonial QA cycle	Pillar 5	Annual working group review; HPCSA report	All roles	HPCSA AIHS accreditation score	≥3/5 within 5 yr*

*All benchmarks are inferential estimates requiring prospective validation. CBE, community-based education; CPD, continuing professional development; OSCE, objective structured clinical examination; QA, quality assurance; THP, traditional health practitioner.

## 2 Materials and methods

### 2.1 Study design and epistemic status

This manuscript reports a three-stage interpretive secondary re-analysis and conceptual synthesis of the documented findings of a prior doctoral investigation (8). No new participants were recruited, no data files were reopened in statistical or qualitative software, and no inferential statistical testing was conducted beyond what was reported in the primary study. Secondary analysis of a consent-cleared HPE dataset is methodologically well-established (16–18).

The framework and roadmap produced here are best understood as an intermediate academic step: a testable proposal for subsequent community co-validation, not an already validated product. All AIHS pillar constructs, roadmap steps, and proposed outcome benchmarks (Table 2) are inferential estimates requiring prospective empirical validation through community co-design, Delphi consensus, and multi-site piloting, constituting the proposed Phase 2 of this research programme.

### 2.2 Theoretical framework

Three intersecting theoretical orientations underpinned the analytical work. Decolonial theory (6, 19) foregrounded the systematic privileging of Western biomedical knowledge over IKS; in this study it functioned not as abstract critique but as a practical reading grid for asking, of each curriculum document and competency score, whose knowledge counts and whose is rendered invisible. Ubuntu philosophy provided the ethical and communitarian foundation (13–15). Cultural humility principles (20) contextualized the curriculum-transformation recommendations, positioning ongoing self-reflection and power-sharing as prerequisites for authentic glocalization. Together, these three orientations guided competency-gap reinterpretation, qualitative thematic re-synthesis, and AIHS Framework construction.

### 2.3 Original dataset

The dataset derives from a doctoral investigation at the UFS School of Clinical Medicine (2020–2022; ethics clearance UFS-HSD2020/1420/2710 and Univ. Bath S20-068). The intern survey yielded a 31.6% response rate (71 of 225 interns invited from approximately 240 eligible; Cronbach’s α = 0.78–0.90). Fifteen educators across all three MBChB phases participated in semi-structured interviews. Documentary analysis covered six institutional curricula and policy documents. The original quantitative and qualitative outputs were generated in standard statistical and qualitative-analysis software during the primary study; the present re-analysis engaged those published outputs rather than the underlying data files. Table 3 summarizes the dataset composition.

**Table 3:** Original dataset composition.

Data type	Description	*n*	Format in primary study	Anonymization
Quantitative	AfriMEDS self-assessment survey of medical interns	71	Descriptive outputs via thesis tables	De-identified
Qualitative	Semi-structured educator interviews	15	Thematic outputs via thesis	Pseudonymized
Documentary	Phase guides, assessment rubrics, policy documents	6	Documentary analysis via thesis	Institutional

### 2.4 Secondary analysis protocol

The secondary analysis followed a four-step protocol that, in plain terms, moves from re-reading the numbers, to re-reading the educator voices, to re-reading the curriculum documents, and finally to drawing these three readings together into the AIHS framework:

Step A: Quantitative competency-gap mapping. Descriptive statistics for each AfriMEDS role were extracted from the proportion of interns responding positively to the summary assessment question for each competency area, as reported in Thesis Table 5.2, with supporting item-level distributions in Thesis Figures 5.2–5.9. An 80% adequacy threshold classified competencies as adequate (≥80%), moderate (50%–79%), or critically deficient (<50%). This threshold was operationalized in the primary study [(8), p.72], where positive composite core-competency scores were classified into three bands (<50%, 50–<80%, and 80–100% of items rated positively on a combined “Strongly agree/Agree” basis), and is consistent with established CBME benchmarks (21, 22).

Step B: Decolonial qualitative thematic re-synthesis. Documented thematic outputs of 15 educator interviews were re-read through a decolonial reading grid (6, 19) following Thomas and Harden’s (18) thematic synthesis procedure. This step produced four interpretive clusters, each mapped onto an AIHS pillar and roadmap step.

Step C: Documentary content re-analysis. An AIHS Document Integration Matrix recorded the presence, absence, or tokenistic representation of each AIHS pillar domain across all six curriculum documents, bounded to the scope of the primary study [(8), Tables 4.1–4.5].

Step D: Convergent synthesis. Findings from all three streams were integrated through complementarity-oriented triangulation (23), subjected to critical decolonial reinterpretation, and mapped onto the five-pillar AIHS Framework. The doctoral thesis is publicly accessible via the University of Bath research repository [(8); see References].

### 2.5 Trustworthiness, reflexivity, and ethics

Credibility was secured by anchoring every interpretive claim in documented primary-study outputs. Transferability was enabled through a thick description of the UFS context. Dependability was supported by an audit trail linking each gap to a corresponding AIHS pillar and roadmap step. Confirmability was enhanced through member checking by all three co-investigators.

The three authors brought deliberately complementary positionalities to the re-analysis. The lead author (N.M.) was an insider faculty member at UFS and the primary doctoral investigator; this proximity afforded contextual depth while creating an interpretive-alignment risk that the reflexive design was built to manage. Professor Priscilla Mpho Jama (P.M.J.; Division of Student Learning and Development, Faculty of Health Sciences, University of the Free State) is a South African health-professions education scholar with institutional knowledge of the UFS MBChB programme and expertise in assessment and student learning, providing an insider-contextual reading of the documentary and competency evidence. Professor Gina Wisker (G.W.; School of Management, University of Bath) is a United Kingdom-based scholar of higher education, doctoral pedagogy, and qualitative inquiry who supervised the primary doctoral study and functioned as a critical outsider (24), interrogating interpretive claims for over-alignment with the lead author’s standpoint. Interpretive inferences were tested across these differing vantage points before being retained. No new ethical clearance was required for this secondary re-analysis of published outputs.

### 2.6 AI disclosure

Generative AI tools (large-language-model assistants, 2025–2026) were used to support manuscript editing and language polishing. All substantive scholarly content, analytical claims, and conclusions are the authors’ own, consistent with Frontiers’ guidelines on AI use in academic writing (25). All figures and table values were subsequently verified by the authors against the primary source data reported in the doctoral thesis (8).

## 3 Results

### 3.1 Quantitative competency gap: an analysis compared to the 80% threshold

**Figure 1:**
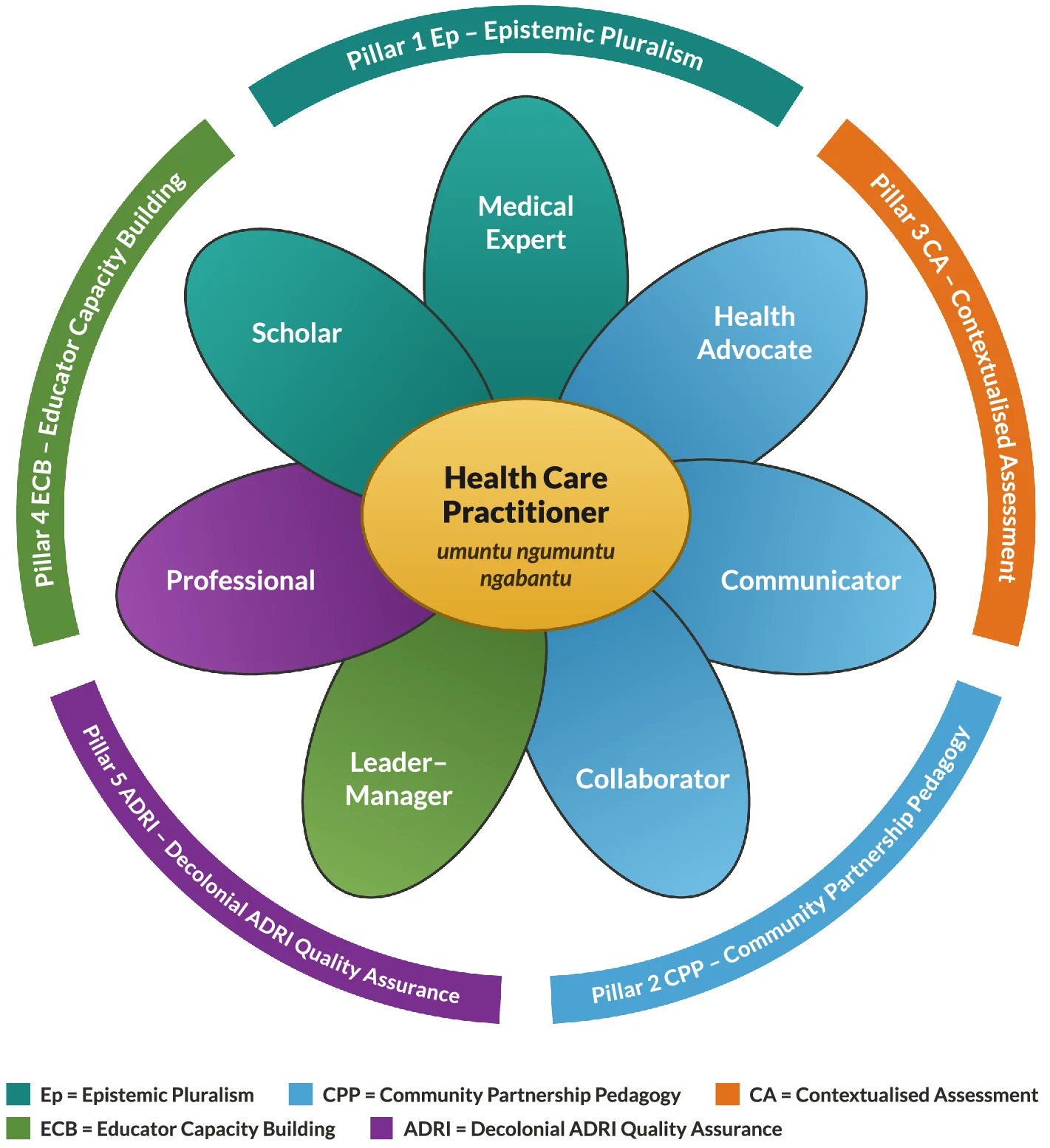
The AfriMEDS–AIHS integrated framework. The seven AfriMEDS physician roles (adapted from the CanMEDS model; Royal College of Physicians and Surgeons of Canada, 2015) are positioned as petals around the central Ubuntu symbol (*umuntu ngumuntu ngabantu*), with the five AIHS pillars forming the outer integrating ring. Color-coding indicates the dominant AIHS pillar for each physician role. Ep, epistemic pluralism; CPP, community partnership pedagogy; CA, contextualized assessment; ECB, educator capacity building; ADRI, decolonial ADRI quality assurance. Copyright © 2015 CanMEDS 2015Physician Competency Framework. Ottawa: Royal College of Physicians andSurgeons of Canada; 2015. Source: https://www.royalcollege.ca/rcsite/canmeds/canmeds-framework-e.

**Figure 2:**
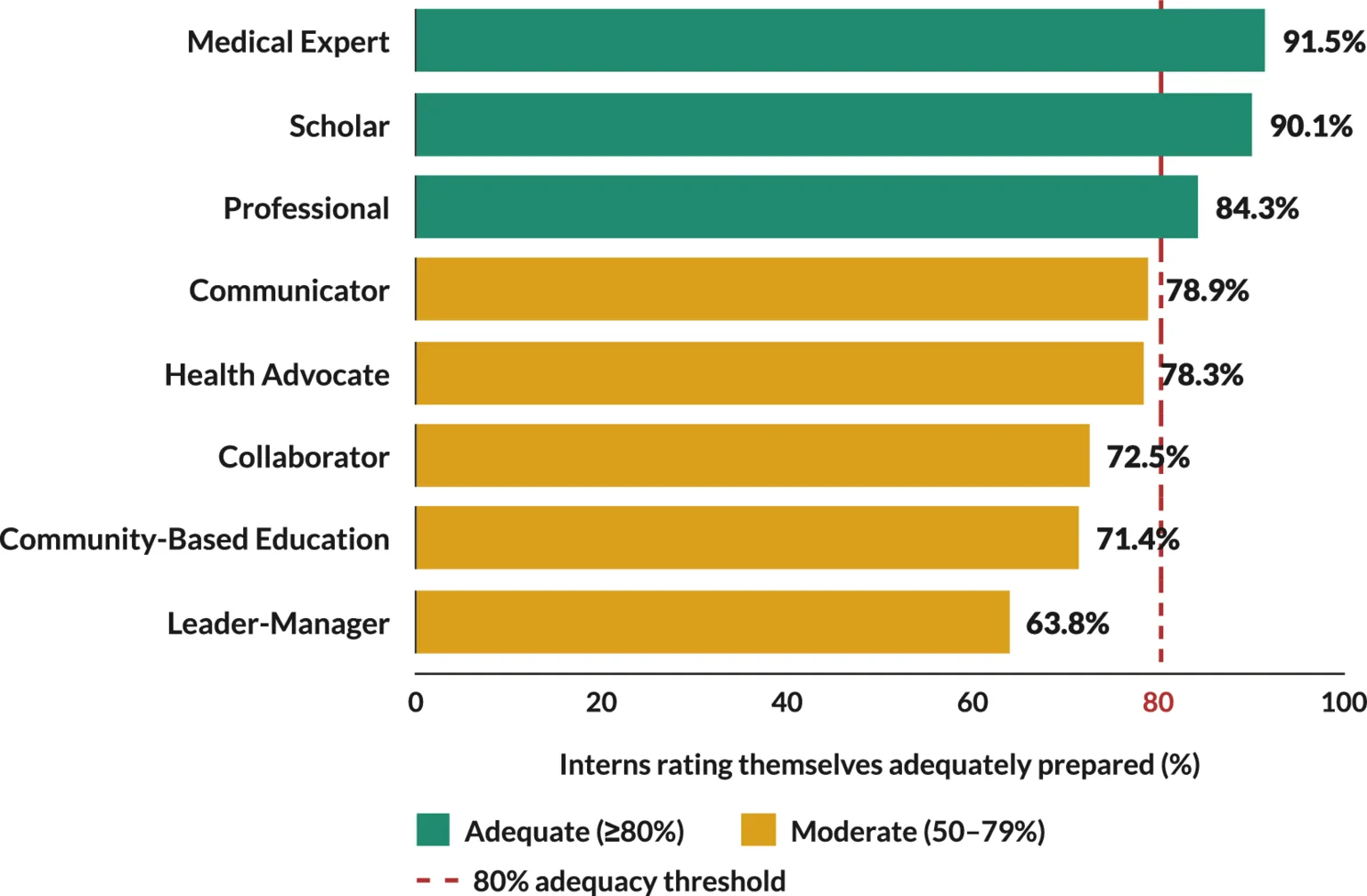
Medical intern self-reported competency preparedness across AfriMEDS domains (*n* = 71). Horizontal bars represent the percentage of interns respondingpositively for each AfriMEDS physician-role domain. The vertical dashed line at 80% denotes the adequacy threshold defined in the primary study (8). Domains below threshold are shaded amber; no domain fell into thecritical band (<50%). Source: secondary analysis of DBA thesisdataset (8, 9), thesis Table 5.2.

Competency-gap analysis revealed a structural dichotomy in intern self-reported readiness. Three AfriMEDS roles met or exceeded the 80% adequacy threshold: Medical Expert (91%), Scholar (90%), and Professional (84%). Five roles fell below: Leader-Manager (63%), Community-Based Education (71%), Collaborator (72%), Health Advocate (78%), and Communicator (79%; Thesis Table 5.2). Deficiencies are concentrated in roles most directly linked to community-responsive practice. The AIHS alignment pattern is diagnostically important: sub-threshold roles map directly onto Pillars 2, 3, and 4, while above-threshold roles concentrate in Pillar 1 (5, 10) (Table 4; Figures 1, 2).

**Table 4:** AfriMEDS competency gap and AIHS pillar alignment.

AfriMEDS role	Adequacy % (*n*) [thesis Table 5.2]	Gap classification	Primary AIHS pillar	Roadmap priority
Medical expert	91% (65/71)	Adequate (≥80%)	Pillar 1: epistemic pluralism	Maintenance
Scholar	90% (64/71)	Adequate (≥80%)	Pillar 1: epistemic pluralism	Maintenance
Professional	84% (59/70)	Adequate (≥80%)	Pillar 5: decolonial QA	Maintenance
Communicator (indigenous lang.*)	79% (56/71) [40%* IKS items]	Moderate/critical*	Pillar 2: community partnership	Priority 1
Health advocate	78% (54/69)	Moderate (50%–79%)	Pillar 2: community partnership	Priority 1
Collaborator	72% (50/69)	Moderate (50%–79%)	Pillar 2: community partnership	Priority 1
Community-based education	71% (50/70)	Moderate (50%–79%)	Pillar 3: contextual assessment	Priority 2
Leader-manager	63% (44/69)	Moderate (50%–79%)	Pillar 4: educator capacity	Priority 2

Source: (8), Thesis Table 5.2, p.114; 80% threshold: (8), p.72. *40% = proportion scoring indigenous-language items positively (Thesis Figure 5.9 narrative, p.113). Percentages are rounded to whole numbers; Figure 2 reports the same values to one decimal place.

### 3.2 Community-based education competency items

**Figure 3:**
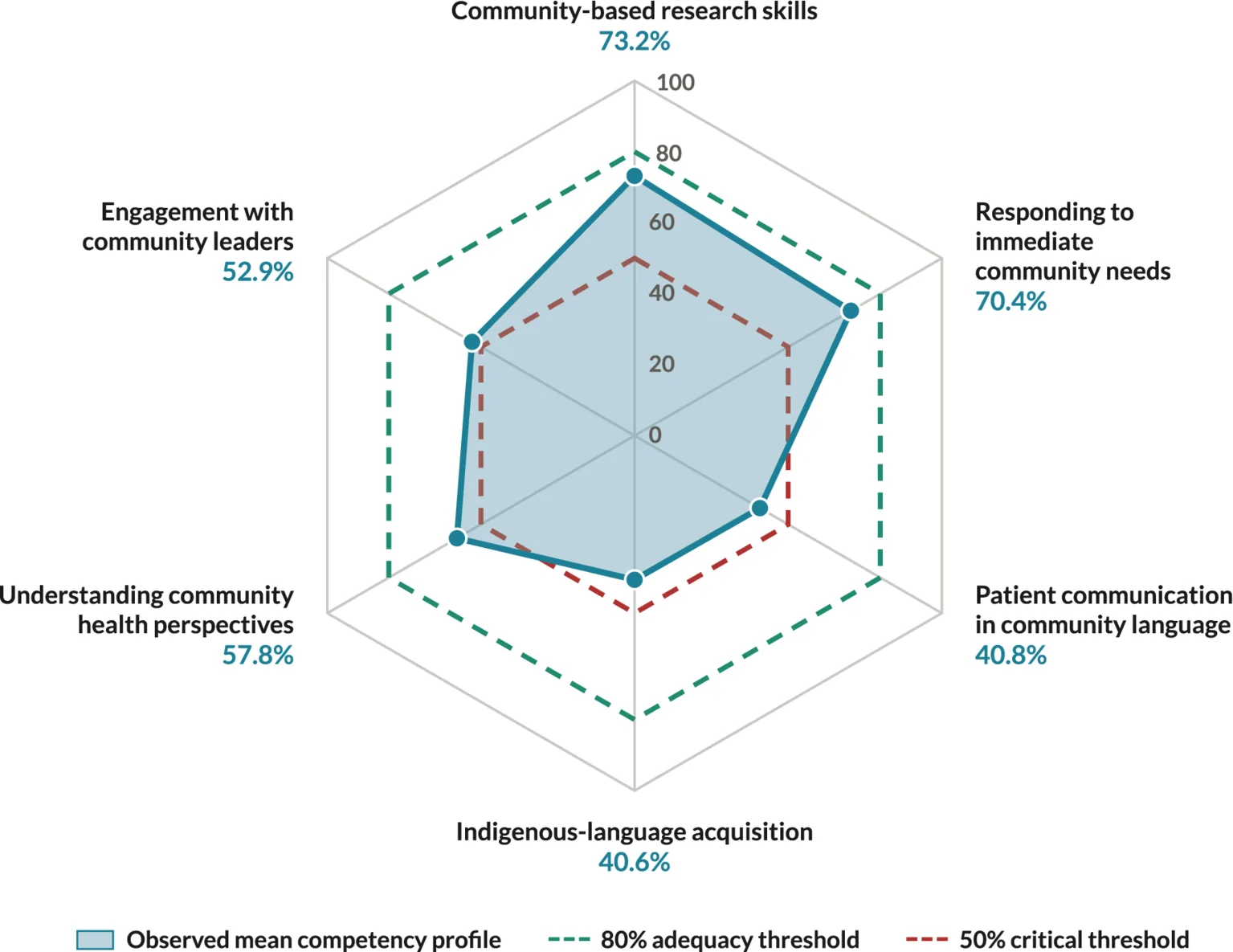
Community-engagement competency (CEC) performance across six domains: radarprofile of item-level adequacy (*n* = 71). Each axis represents one of the six CECs, labelled by competency name. The inner dashed hexagon marks the 50% critical threshold and the outer dashed hexagon the 80% adequacy threshold. The shaded area represents the observed competency profile. Critical deficits (<50%) are visible in patient communication (40.8%) and indigenous-language acquisition (40.6%). Source: secondary analysis of DBA thesis dataset (8).

All community-based education (CBE) competency items fell below the 80% adequacy threshold [(8), Figure 5.9, p.113; narrative pp.113–114]. The items scoring most critically were those relating to indigenous-language communication and community-language interaction, at 40.8% and 40.6% positive response respectively. The highest-scoring CBE item was “understand community perspective on their healthcare needs” at 73.2%. A mean well below 80% across all CBE competency items indicated structural rather than incidental inadequacy (Table 5; Figure 3).

**Table 5:** Community-based education competency items.

CBE competency item (Thesis Figure 5.9)	AfriMEDS role	Adequacy (%)	Gap classification	AIHS pillar/step
Understand community perspective on healthcare needs	Health advocate	~73.2%^†^	Moderate	Pillar 1/Step 1
Know how to respond to immediate community needs	CBE/health advocate	~70.4%^†^	Moderate	Pillar 2/Step 3
Know how to interact with community leaders	Leader-manager	~57.8%^†^	Moderate	Pillar 4/Step 4
Know how to conduct community-based research	Health advocate	~52.9%^†^	Moderate	Pillar 2/Step 2
Communicate with patients in community language	Communicator	40.8%	Critical (<50%)	Pillar 2/Step 2
Learn basic language relevant to community	Communicator	40.6%	Critical (<50%)	Pillar 2/Step 2

Source: (8), Figure 5.9, p.113 and the corresponding appendix item tables; narrative summary pp.113-114. Values are item-level positive-response proportions (Agree + Strongly agree) taken directly from the primary study. Items are listed in descending order of adequacy; no item reached the 80% threshold.

### 3.3 Documentary evidence: IKS and Ubuntu absence

Re-analysis of six institutional documents using the AIHS Document Integration Matrix produced a consistent finding: IKS, traditional medicine, THP-collaboration protocols, and Ubuntu ethics are either entirely absent or represented tokenistically. We do not present this as a surprising discovery; given the framework’s documented origins, a degree of absence was to be expected. Its analytical value lies elsewhere, in converting a theoretically predicted absence into documented, document-by-document evidence, and so establishing the empirical baseline against which the educator testimony in Section 3.4 and the roadmap in Section 3.6 are calibrated. This finding is bounded by the scope of the primary study: it characterizes the six documents analyzed in Mofolo (8), Tables 4.1–4.5; documents outside this original study remain beyond its scope. As Mnguni (10) demonstrates through curriculum-ideology analysis, this absence reflects an ideological configuration that defaults to scholar-academic and social-efficiency orientations, structurally excluding social-reconstructionist knowledge and rendering curriculum transformation a matter of epistemic justice, not merely resource allocation (Table 6).

**Table 6:** AIHS document integration matrix: presence, absence, or tokenistic representation across six curriculum documents.

AIHS pillar domain	HPCSA accred. 2017	Phase (I–III)	UG rule book	2019 MBChB summit	Overall status
IKS integration	Absent	Absent	Absent	Absent	Complete absence
THP collaboration protocols	Absent	Absent	Absent	Absent	Complete absence
Indigenous-language assessment	Tokenistic	Absent	Absent	Absent	Critically deficient
Health advocate in early phases	Absent	Present (Phase III, once)	Absent	Tokenistic	Structurally marginalized
Ubuntu/community ethics	Absent	Absent	Absent	Tokenistic	Complete absence
CBE/COPC explicit outcomes	Tokenistic	Tokenistic/moderate	Tokenistic	Moderate	Tokenistic across most

Source: secondary analysis of (8), Tables 4.1–4.5. Absent = zero instances of substantive integration; Tokenistic = limited mention without assessment accountability. The six documents comprise the 2017 HPCSA accreditation report, the three phase guides (Phases I-III), the undergraduate rule book, and the 2019 MBChB curriculum review summit.

### 3.4 Educator perspectives: four thematic clusters

All qualitative evidence in this section derives exclusively from the fifteen educator interviews documented in the primary study. The original intern survey instrument captured closed competency ratings only and contained few open-ended items; there is therefore no intern qualitative dataset to carry into the thematic synthesis, and we make this boundary explicit so that the interns’ recession from the quantitative analysis (3.1–3.2) into the background of the qualitative analysis reads as a property of the source data rather than an analytic oversight. Following Thomas and Harden’s (18) thematic-synthesis procedure, the documented thematic outputs were re-read through a decolonial analytical lens to generate interpretive constructs that move beyond the original evaluation framing. The four clusters set out below are developed through meaning rather than measurement: each is presented through what it signified for the educators who voiced it and what it implies for curriculum design, with the underlying coding record retained only as a corroborating audit trail and reported, for transparency, in the endnote to this section.

#### 3.4.1 Theme 1: structural barriers and the over-stretched supervisor

The most insistent thread running through the educator accounts was not a deficit of conviction but a deficit of capacity. Educators who plainly valued community-oriented training described themselves as working within a programme whose architecture left no room to deliver it. Their language was the language of erosion: too few staff, too many students, too little time, and clinical exposure that had thinned to the point where the community-facing competencies could not be modelled, let alone supervised. One educator distilled the problem to its constituent parts, identifying “*lack of staff*, *an increasing number of students, lack of time and limited clinical exposure*” as the conditions that would first have to be addressed before AfriMEDS could be implemented in any meaningful way and internship preparation genuinely strengthened.

Yet the educators were not passive within these constraints, and it would misrepresent the data to portray them only as victims of structure. Several described improvised, unfunded efforts to keep community-oriented teaching alive: informal mentorship at rural sites, the personal cultivation of relationships with clinics, and the quiet protection of placement experiences they valued even when the timetable did not reward them. These grassroots initiatives matter analytically, because they demonstrate that the will to teach community-responsive medicine already exists within the faculty; what is missing is the structural recognition, time, and legitimacy that would convert individual goodwill into a reliable, assessable curriculum. Read decolonially, this cluster reveals an institutional design failure rather than a motivational one, and the distinction matters for where remedy is sought. The educators had themselves been formed in curricula that never treated community medicine as a distinct, teachable body of expertise; the knowledge they were now expected to transmit had never been systematically recognised as knowledge within the structures that trained them. The shortage they described was thus doubly structural: a shortage of bodies in the present, layered over a historical shortage of legitimacy for the very domain they were being asked to teach. A faculty cannot reliably pass on an expertise that its own formation declined to name. Mapped to Pillar 4 (educator capacity building) and Roadmap Step 4, the theme calls for dedicated AIHS faculty-development provision that addresses the competency deficit and the workload conditions together, since training offered without protected time to attend it simply reproduces the constraint it was meant to relieve.

#### 3.4.2 Theme 2: indigenous knowledge as both risk and aspiration

A second, more ambivalent theme emerged whenever educators spoke about indigenous knowledge. They affirmed its cultural and clinical relevance readily, and several spoke of it as something the curriculum ought to honour; yet in almost the same breath they confessed that they did not know how to teach or assess it, because it had been absent from their own training and remained absent from the documents that governed their teaching. The aspiration and the apprehension were inseparable: educators wanted to integrate community-embedded knowledge but experienced its integration as professionally risky, an undertaking for which they had neither a curricular mandate nor a pedagogical method.

This educator-level testimony aligns precisely with the documentary finding reported in Section 3.3, where IKS, THP collaboration protocols, and Ubuntu-aligned ethics were found to be absent or merely tokenistic across all six institutional documents. The convergence is analytically significant: the same epistemic absence appears at two levels of the system at once, in the official architecture of the curriculum and in the formation of the people expected to enact it. Mnguni’s (10) curriculum-ideology analysis supplies the explanation, showing that a framework dominated by scholar-academic and social-efficiency orientations produces educators who are, in effect, epistemically monolingual in biomedicine; fluent in one knowledge tradition and unequipped in the other not by disposition but by design. Reversing this requires more than exhortation. It requires deliberate epistemic-pluralism infrastructure: co-educator agreements with THPs, IKS reading embedded from the first phase of study, and faculty workshops co-facilitated by community knowledge-holders. Operationalized in Roadmap Step 2 and mapped to Pillar 1, the theme reframes IKS not as an optional enrichment but as legitimate curricular content that the institution must actively underwrite.

#### 3.4.3 Theme 3: community-based placements as constraint and transformative potential

Educators held CBE in evident regard, describing it as among the most formative experiences the programme offered, while simultaneously portraying it as under-resourced and pedagogically incomplete. The tension was not incidental but structural, and it surfaced most clearly when educators contrasted what placements achieved with how they were provisioned. One educator, describing the interprofessional Trompsburg rural training platform, captured the transformative end of the spectrum:

*“We have got three sessions on interprofessional training where they need to make a video and things like that and they go to Trompsburg together, all the Allied Health Nursing students as well as the medical students for a week, and they work together; they have got their own programmes, projects. They go to schools, they go to old age homes to give presentations. That’s also assessed, and they have got outcomes for that, and they get marks for that.”*

Against that account stood the candid recognition of “*limited resources and community-based training facilities,*” and of practical constraints that pushed institutions toward the expedient of placing, in one educator’s phrase, “*X number of students*” at sites without adequate support. These are not abstractions: the platforms in view are small rural Free State district facilities such as Trompsburg, serving predominantly Sesotho- and Afrikaans-speaking rural households, where a single overstretched clinic may host a cohort with limited supervisory cover and no formal indigenous-language scaffolding. The decisive analytic point, however, is not merely that placements are under-resourced; it is that the elements which would make them authentically glocal, structured visits to THPs and deliberate indigenous-language learning, are absent from the design logic of placements altogether. They were not crowded out by scarcity; they were never built in. Mapped onto Pillar 2 and Roadmap Step 3, the theme calls for THP co-facilitator roles with defined pedagogical responsibilities, indigenous-language learning embedded as a standard, credited placement component, and community-based logbooks redesigned to carry AIHS reflection portfolios, so that what the placement values becomes what the placement is built to deliver.

#### 3.4.4 Theme 4: assessment as epistemic gatekeeper

The final theme located the deepest leverage point in the system: assessment. Educators observed that culturally responsive community competencies remained largely outside formal summative assessment, and that this exclusion rendered them effectively invisible to the students who were supposed to acquire them. The mechanism they described was simple and unsentimental: students invest effort where marks are awarded, so competencies that carry no marks attract no effort. One educator named the casualties precisely:

*“Professionalism and whether they’re good communicators, whether they can have good leadership skills, and collaborate well with the team, and whether they will be good at working in a healthcare environment, those are definitely gaps, which we are not assessing, currently.”*

Another traced the same failure to an assessment architecture that rewards siloed recall over integration:

*“Stop treating your content as if you are dealing with silos, you need to be able to integrate… We never get the opportunity to follow up on the skill of integrating; I don’t think it’s emphasised.”*

The competencies these educators identified as unassessed, collaboration, communication, and community-oriented professionalism, are precisely the AfriMEDS roles most closely bound to indigenous-knowledge integration, which gives the theme its decolonial edge. What is at stake is not an oversight to be corrected by adding a station, but a structural property of an assessment system organised around biomedical content recall. As Pangaro and Ten Cate (21) observe, a competency that is not assessed is not merely left untaught; it is progressively unmade, its absence reproduced silently across successive cohorts until it ceases to register as a gap at all. Mapped onto Pillar 3 and Roadmap Step 5, the theme calls for IKS-aligned portfolio tasks, indigenous-language OSCE stations, and Ubuntu-ethics assessment scenarios, so that the instruments of judgement begin to value what the framework claims to hold dear (Table 7).

**Table 7:** Educator theme reference matrix: AIHS pillar, thematic cluster, and narrative salience (*n* = 15).

AIHS pillar	Thematic cluster	Narrative salience (thesis Ch. 4)
Pillar 4: educator capacity	Theme 1: structural barriers and the over-stretched supervisor	A dominant concern across the educator interviews; staff shortage and CBME-preparation constraints were among the most frequently and forcefully raised issues.
Pillar 1: epistemic pluralism	Theme 2: IKS integration as risk and aspiration	Recurrent; educators repeatedly returned to the absence of IKS from their own formation and from curriculum documents.
Pillar 2: community partnership	Theme 3: CBE constraints and transformative potential	Prominent; community-based placement featured strongly wherever educators discussed assessment and practical training.
Pillar 3: contextual assessment	Theme 4: assessment as epistemic gatekeeper	Predominant; assessment was the single most heavily discussed domain, and the gap in assessing non-technical competencies was raised consistently.

Source: Mofolo (8), Chapter 4 thematic interview analysis. Salience is reported narratively; the underlying coding frequencies are given in the endnote.

Taken together, the four clusters describe a single, self-reinforcing system rather than four separate problems. A faculty under-resourced and under-prepared (Theme 1) cannot reliably transmit a knowledge tradition its own formation never legitimized (Theme 2); placements that might carry that knowledge are not designed to do so (Theme 3); and an assessment regime that ignores the relevant competencies removes any incentive to acquire them (Theme 4). It is this interlocking quality that the AIHS Integration Framework and the six-step roadmap are constructed to disrupt, by intervening at each point in the cycle simultaneously rather than at any one in isolation. Table 7 maps each cluster to its corresponding AIHS pillar and indicates its salience within the educator accounts.

#### 3.4.5 Endnote to section 3.4 (audit trail)

The interpretive account above is anchored in the documented coding record of the primary study, summarised here for transparency rather than carried in the body text. In the primary study’s broad key-area coding [(8), Table 4.7, p. 86], six key areas were coded across 283 citations in total: Assessment of competencies (130 citations, 46%), Challenges (40, 14%), Support required from management (35, 12%), Needs for effective assessment of students (27, 9%), Gaps (26, 9%), and Best practices (25, 8%). The four areas discussed here are those bearing directly on the thematic clusters. The structural constraints discussed under Theme 1 recur principally within the Challenges and Support-from-management areas; the indigenous-knowledge concerns of Theme 2 appear across the Challenges and Gaps areas; the placement evidence of Theme 3 sits within the Assessment-of-competencies and Challenges areas; and the assessment evidence of Theme 4 is concentrated in the Assessment-of-competencies area, with the specific unassessed-competency gaps catalogued in Mofolo (8), Table 4.9. These figures are reported as a corroborating audit trail; consistent with thematic synthesis (18), the analysis above is organised by interpretive salience rather than by frequency of coding.

### 3.5 The AIHS integration framework: five pillars

Convergent synthesis produced the AIHS Integration Framework, comprising five mutually reinforcing pillars. These are conceptual and inferential constructs requiring prospective community validation (Figure 1):

- Pillar 1: epistemic pluralism. Recognition of IKS, THP knowledge, and Ubuntu wisdom as legitimate medical knowledge alongside biomedicine. Epistemic pluralism is not the uncritical adoption of either tradition: it holds biomedicine and indigenous knowledge as co-equal interlocutors, each accountable to evidence, safety, and the patient’s good. Authentic integration must therefore engage real points of friction, for example, the standardization of herbal preparations and dosing, the management of potential interactions with prescribed medicines, and the navigation of genuinely conflicting therapeutic advice, rather than romanticising traditional practice as a frictionless alternative. Naming these complexities is itself part of taking indigenous knowledge seriously as knowledge. Motivated by universal sub-threshold IKS performance and documentary absence.
- Pillar 2: community partnership pedagogy. Deliberate curriculum structures for THP co-facilitated placements and credited indigenous-language modules. Motivated by indigenous-language adequacy at 40% (Thesis Figure 5.9, p.113).
- Pillar 3: contextualized assessment. AIHS-aligned instruments including community-diagnosis OSCEs and indigenous-language rubrics embedded in summative assessments. Motivated by educator Theme 4.
- Pillar 4: educator capacity building. Structured faculty-development programmes equipping educators with AIHS knowledge and culturally humble pedagogy. Motivated by educator-reported inadequate CBME preparation [(8), Chapter 4, Challenges key area].
- Pillar 5: decolonial quality assurance. Integration of AIHS alignment, community satisfaction, and Ubuntu-ethics indicators within HPCSA accreditation. Motivated by the absence of community-responsive quality indicators.

### 3.6 The six-step AfriMEDS–AIHS implementation roadmap

**Figure 4:**
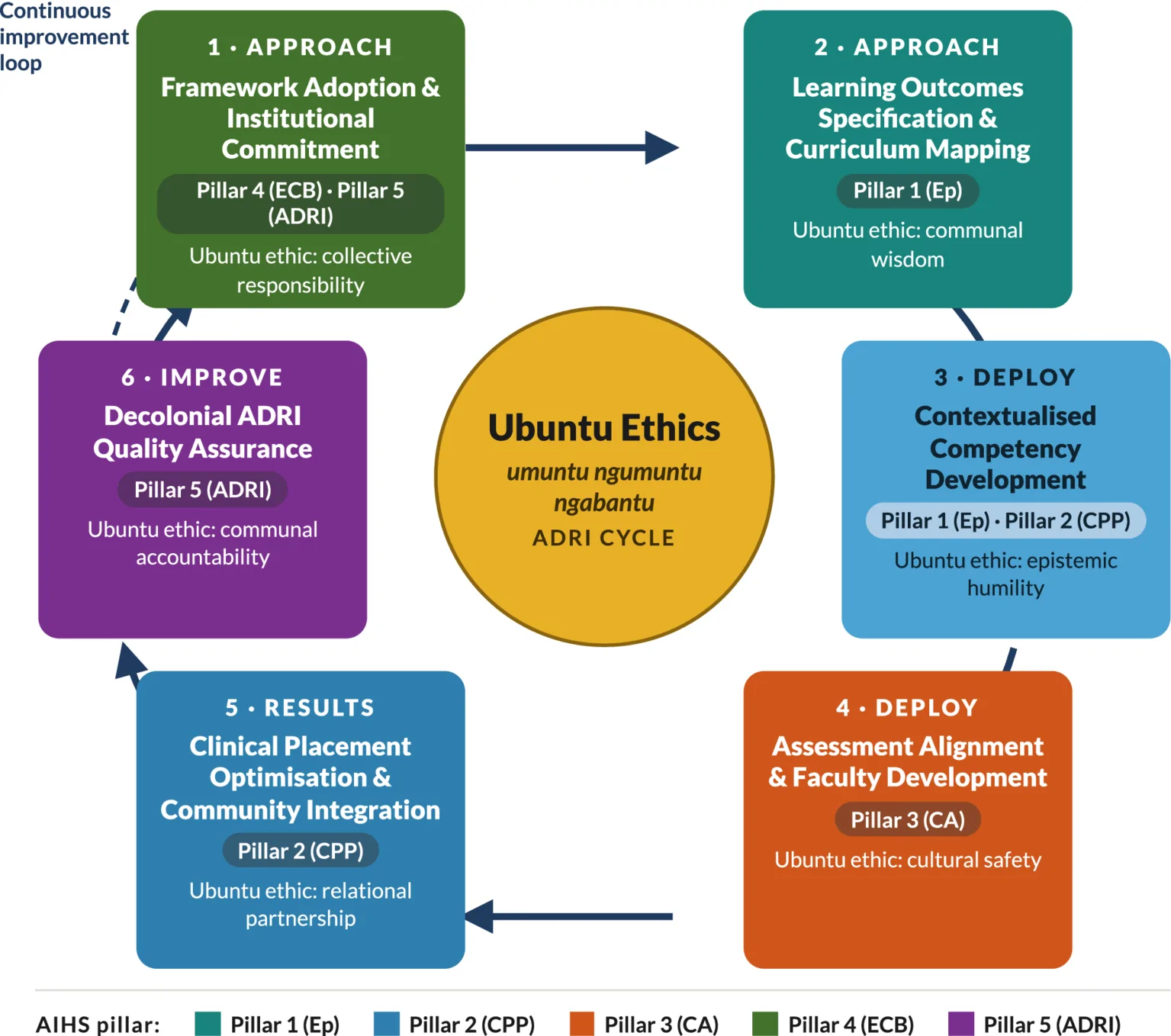
The six-step AfriMEDS–AIHS implementation roadmap as a continuous ADRI cycle. Steps 1-2 (Approach): decolonial curriculum audit and epistemic pluralism co-design; Steps 3-4 (Deploy): community-based learning and language restructuring, and faculty development; Step 5 (Results): AIHS-aligned summative assessment integration; Step 6 (Improve): decolonial quality assurance and community feedback integration. Each step is labeled with its primary AIHS pillar linkage and the Ubuntu ethical principle that grounds it. Arrows indicate the iterative, nonlinear nature of the cycle. Source: Adapted from the AfriMEDS implementation guidelines framework (8, 9).

The six-step roadmap operationalizes the five AIHS pillars as a sequenced institutional change process for HPCSA-accredited medical schools, transferable to comparable Global South HPE programmes. All outcome indicators are inferential and require prospective longitudinal validation (Figure 4).

#### 3.6.1 Step 1: Decolonial curriculum audit and epistemic mapping (Pillar 5; Ubuntu ethic:collective responsibility)

Establish an evidence-based baseline of current IKS, THP, Ubuntu, and indigenous-language integration across all curricular phases. Conduct a gap analysis of the AIHS documentary resources, including phase guides, assessment rubrics, and policy documents. Administer the AfriMEDS CEC Survey to intern cohorts annually. Produce a gap-analysis dashboard for the curriculum committee. Engage community representatives in validating findings.

#### 3.6.2 Step 2: Epistemic pluralism curriculum co-designworkshop (Pillars 1 and 2; communal wisdom)

Co-design IKS modules, THP-partnership activities, Ubuntu-ethics cases, and indigenous-language curricula with THPs, community educators, and faculty. Establish a Glocalization Working Group whose recommended composition is deliberately cross-constituency: registered Traditional Health Practitioners, community elders and patient representatives, indigenous-language and cultural advisors, clinical and basic-science faculty, curriculum and assessment managers, and student representatives, co-chaired by a community knowledge-holder and a curriculum lead so that authority over design is genuinely shared rather than nominal. Develop indigenous-language communication objectives for each MBChB phase. Map IKS activities to the AfriMEDS roles of Communicator, Collaborator, and Health Advocate.

#### 3.6.3 Step 3: Contextualised community-basedlearning and language restructuring (Pillar 2; relational partnership)

Redesign community placements to include structured THP co-facilitation, indigenous-language learning, and Ubuntu-relational learning outcomes. The severity of the language finding, only 40% of interns felt prepared to communicate with patients in an indigenous language, demands a structural rather than a cosmetic response. Informal “*conversation hours*” alone are insufficient to the scale of a 60% preparedness deficit and risk repeating the very tokenism this framework critiques. We therefore propose mandatory, credit-bearing indigenous-language modules, scaffolded across the MBChB phases and assessed summatively: foundational clinical vocabulary and greetings in the relevant regional language(s) in the early phases; history-taking and explanation-of-diagnosis competence in the clinical years; and integrated, assessed indigenous-language patient encounters during community-based placements. These modules should articulate with national language policy and, where feasible, with university language units, so that proficiency is built systematically rather than left to individual initiative. Establish formal THP co-facilitator roles with defined pedagogical responsibilities and appropriate remuneration. Redesign CBE logbooks to include AIHS reflection portfolios.

#### 3.6.4 Step 4: Faculty development programme for AIHS-competent educators (Pillar 4;epistemic humility)

Build educators’ capacity in AIHS content knowledge, culturally humble pedagogy, and Ubuntu-relational teaching practices. The framework must reconcile its own ambition with the staff shortages and over-stretched supervisors documented in Theme 1; proposing resource-intensive faculty development for an environment already short of time and people would otherwise be self-defeating. We therefore frame Step 4 as phased and resource-realistic. The headline target of 24 contact hours per year is an aspiration for mature implementation, not an entry requirement: programmes may begin with a low-cost core, short integrated sessions within existing Continuous Professional Development (CPD) and staff-meeting time, THP and community knowledge-holders invited as guest facilitators rather than salaried appointments, and peer-learning communities that distribute teaching load across the three MBChB phases. More ambitious provision can then be funded incrementally through HPCSA-aligned CPD accreditation, existing teaching-development grants, and the institutional and national funding streams identified in Section 4.4, so that faculty development scales with secured resources rather than presupposing them. Align all provision to HPCSA CPD requirements.

#### 3.6.5 Step 5: AIHS-aligned summative assessment integration (Pillar 3; cultural safety)

Embed AIHS and Ubuntu-competency assessment within formal summative assessments. Design community-diagnosis OSCEs that include THP consultation scenarios and indigenous-language tasks, develop portfolio rubrics for cases involving patients who use traditional medicine, and include Ubuntu-relational ethics scenarios in professional-ethics assessment stations. Submit revised assessment blueprints to HPCSA for accreditation review.

To make this tangible, consider one worked example of a community-diagnosis OSCE station. A standardized patient presents with poorly controlled hypertension and discloses that she is also taking a herbal remedy prescribed by a traditional health practitioner. A second role-player, a THP or a trained actor briefed in the role, is present. The candidate is required, within the station, to take an accurate history in the patient’s indigenous language (or to work appropriately with an interpreter while demonstrating respectful language use), to elicit the traditional treatment without dismissiveness, to reason safely about possible interactions between the herbal preparation and the prescribed antihypertensive, and to negotiate a shared management plan that respects the patient’s relationship with the THP while safeguarding clinical safety. Scoring is against a co-developed rubric with weighted domains: clinical reasoning and safety; indigenous-language and communication competence; cultural humility and Ubuntu-relational conduct (assessed through observable behaviours rather than self-report); and collaborative engagement with the THP. Rubrics are co-designed with community members and THPs, triangulated with simulated-patient feedback, and calibrated across sites through examiner training, so that cultural humility is assessed as demonstrated practice and not reduced to a checkbox.

#### 3.6.6 Step 6: Decolonial quality assurance and continuousimprovement (Pillar 5; communal accountability)

Institutionalize AIHS alignment as a formal quality indicator within the programme’s ADRI quality assurance (QA) improvement cycle and HPCSA accreditation submissions. Define AIHS Outcome Indicators (Table 2) with proposed benchmarks for annual monitoring. Embed community-satisfaction and THP-partnership data in programme-review cycles. Report on AIHS alignment progress in HPCSA accreditation self-evaluation reports.

## 4 Discussion

### 4.1 From compliance to glocalization: the central argument

The AfriMEDS evaluation data reveal a compliance-to-glocalization gap that is both quantifiable and deeply epistemic. Three roles; Medical Expert, Scholar, and Professional, achieved adequacy precisely because they align with the biomedical technical knowledge that has dominated South African medical curricula since the colonial era. The five sub-threshold roles, Leader-Manager, CBE, Collaborator, Health Advocate, and Communicator, are precisely those requiring indigenous-language proficiency, THP engagement, community diagnosis, and Ubuntu-relational leadership.

This pattern is not incidental; it is structural. Mnguni (10) demonstrates that the AfriMEDS framework, as currently designed, is dominated by scholar-academic and social-efficiency orientations which structurally crowd out social-reconstructionist content, including IKS, Ubuntu ethics, and community-partnership pedagogy. This ideological configuration explains why the sub-threshold roles are precisely those requiring epistemic pluralism, indigenous-language proficiency, and THP engagement: the curriculum ideology that shaped AfriMEDS did not prioritize these competencies as knowledge, but only as aspirational policy language. Authentic glocalization therefore requires a deliberate ideological reorientation; centering IKS and Ubuntu as epistemically equivalent foundations of medical knowledge, while retaining, not displacing, the biomedical competence the same data show the programme already delivers well.

This argument extends beyond the South African context. McKenzie-White et al. (5) identified parallel glocalization deficits in CBME implementation across East Africa; Ajemba et al. (4) found similar patterns in West African HPE. de Sousa Santos’ (6) concept of the epistemology of the South; foregrounding knowledge historically marginalized by colonial education systems, underpins the case for treating IKS and Ubuntu not as contextual supplements but as foundational curriculum elements.

### 4.2 The integrated change pathway: why sequence matters

The six-step roadmap is explicitly sequenced because the sequence matters. An audit without co-design produces diagnoses without remedies. Co-design without faculty development produces curricula that educators cannot teach. Summative assessment without prior curriculum redesign assesses competencies that have not been developed. Quality assurance without community-satisfaction data perpetuates institutional self-referentiality. The ADRI quality assurance cycle is the governance architecture that enables iteration: each cycle begins with an approach informed by the decolonial audit, deploys it through co-designed faculty and student activities, generates results through AIHS-aligned assessment, and returns to improvement through the annual Working Group review and HPCSA reporting process. This sequenced integration addresses the implementation failure mode identified by Ajemba et al. (4): *ad hoc* CBME interventions that address isolated elements without disrupting the structural logic of the existing curriculum.

### 4.3 Positioning in the international literature

The AfriMEDS–AIHS framework makes three contributions to the international HPE glocalization literature. First, it operationalizes decolonial theory (6, 19, 29) as a practical curriculum architecture rather than a political aspiration. Second, it provides an empirically anchored, pillar-structured roadmap that is transferable: any programme with access to AfriMEDS (or an equivalent CBME framework) and a community-engagement audit methodology can enter the roadmap at Step 1. Third, the Glocalization Working Group model, in which THPs, community representatives, and clinical faculty jointly design, facilitate, and evaluate curriculum, operationalizes Boelen and Woollard’s (26) social-accountability principle at the institutional level. Mnguni (10) adds an important curriculum-ideology dimension not fully articulated in either the doctoral thesis or the Beyond Compliance article.

### 4.4 Policy and practice implications

A transferability claim of this scope requires an explicit warrant, since the empirical base is a single South African institution. The warrant is structural rather than statistical. The mechanisms documented at UFS, a curriculum ideology that privileges biomedical recall, an assessment architecture that renders non-technical competencies invisible, faculty formed without legitimacy for community medicine, and placements never designed to carry indigenous knowledge, are not idiosyncratic to one campus. They are the predictable local expression of a CanMEDS-derived competency model exported, with limited epistemic adaptation, across the Global South, and the comparative CBME literature reports the same failure pattern in East and West African settings (4, 5). What transfers, therefore, is not the UFS dataset but the diagnostic logic and the staged response: any programme built on an imported competency framework can run the Step 1 audit against its own documents and competency data, and will, we contend, surface structurally similar deficits to which the same six-step sequence can be adapted. The roadmap is offered in that spirit, as a transferable method requiring local re-grounding, not a finding generalized beyond its evidence.

Three priority shifts follow. For the HPCSA, the decolonial QA indicators proposed in Step 6 and Table 2 provide a basis for a revised AIHS-alignment dimension within the Medical and Dental Professions Board accreditation standards. For medical schools, the roadmap is deliberately phased so that it begins in low-resource settings: Steps 1 and 2 require commitment rather than capital, since an audit and a co-design workshop can be mounted within existing structures, while Steps 3–6 can be sequenced as institutional and external resources allow. For funders and national authorities, ring-fenced support for THP partnerships, credited indigenous-language training, and community-led evaluation would convert these from optional add-ons into core, fundable graduate competencies. The framework can be adapted by any national medical-education system in the African Union and the wider Global South that has a CBME-derived competency framework and aims to integrate IKS and community partnerships (1, 11).

### 4.5 Limitations and reflexivity

Six limitations require acknowledgment. First, the secondary analysis is bounded by the scope of the primary study, drawn from a single South African institution; transferability requires contextual adaptation, as argued in Section 4.4. Second, the framework is inferential: every AIHS pillar, roadmap component, and outcome benchmark is a proposal pending community co-validation. Third, the intern survey’s 31.6% response rate and its cross-sectional design, and the achieved sample was not benchmarked against the demographic profile of the eligible cohort, so representativeness cannot be assumed. Fourth, the qualitative sample (*n* = 15) is purposive; no saturation claim is made. Fifth, the documentary sample (*n* = 6) represents significant but not exhaustive institutional documents. Sixth, as co-architects of the primary study, the authors face inherent interpretive-alignment risks managed through reflexive triangulation (Section 2.5). Future research should include a national Delphi consensus process with THPs, HPE educators, and community representatives, followed by multi-site piloting and longitudinal outcome evaluation.

## 5 Conclusion

Glocalizing AfriMEDS is a deliberate educational and political act, a move from policy compliance toward epistemic freedom. The AIHS Integration Framework and six-step implementation roadmap provide a practically oriented, theoretically grounded pathway for HPCSA-accredited medical schools and Global South HPE programmes to produce graduates who are globally informed, clinically competent, and communally accountable. In operational terms, that pathway is concrete rather than merely aspirational: it begins with a decolonial curriculum audit (Step 1), proceeds through community co-design and contextualized, credited language and placement restructuring (Steps 2–3), builds the faculty capacity and assessment instruments that make the new competencies real (Steps 4–5), and closes the loop through community-anchored quality assurance (Step 6). The framework demonstrates that CBME standards and indigenous knowledge systems are not in tension, and that Ubuntu philosophy and global competency frameworks are not competing claims but complementary foundations. Integrated thoughtfully through the five AIHS pillars, they form the basis of a medical-education system capable of producing the community physicians Africa needs. Implementation begins with an audit. The roadmap starts here.

### 5.1 Recommendations

- For the HPCSA and national policy: revise AfriMEDS accreditation criteria to require demonstrable AIHS and Ubuntu integration in learning outcomes, assessment instruments, and quality-assurance cycles, and recognise community-advisory participation as formal accreditation evidence.
- For medical schools: adopt the six-step roadmap, beginning with the low-cost Steps 1–2 and prioritising Pillars 2 and 3, where the quantitative deficits are most severe.
- For educators and clinical departments: embed credited indigenous-language and cultural-humility assessment into workplace-based instruments, and formalize THP partnerships at accredited training platforms.
- For funders and institutional leaders: ring-fence support for educator capacity building, indigenous-language training, and community-led programme evaluation.
- For researchers: prospectively evaluate the roadmap across multiple African schools, with THPs, patients, and community leaders positioned as co-researchers rather than subjects.

## Data Availability

No new data were generated for this study. This work is a secondary analysis of previously published outputs, namely the doctoral thesis Mofolo (8), publicly accessible through the University of Bath research repository at https://researchportal.bath.ac.uk/en/studentTheses/evaluation-of-the-implementation-and-assessment-of-afrimeds-physi, and Mofolo, Jama and Wisker (9), doi 10.1007/s10459-025-10474-z. All data analysed are contained within those published sources. Further enquiries may be directed to the corresponding author.
